# The interferon stimulated gene viperin, restricts *Shigella. flexneri in vitro*

**DOI:** 10.1038/s41598-019-52130-8

**Published:** 2019-10-30

**Authors:** K. J. Helbig, M. Y. Teh, K. M. Crosse, E. A. Monson, M. Smith, E. N. Tran, A. J. Standish, R. Morona, M. R. Beard

**Affiliations:** 1 Department of Physiology, Anatomy and Microbiology, La Trobe University, Melbourne, Victoria Australia; 2 Department of Molecular and Biomedical Science, Research Centre for Infectious Diseases, University of Adelaide, Adelaide, South Australia

**Keywords:** Antimicrobial responses, Innate immunity, Microbiology, Bacteriology

## Abstract

The role of interferon and interferon stimulated genes (ISG) in limiting bacterial infection is controversial, and the role of individual ISGs in the control of the bacterial life-cycle is limited. Viperin, is a broad acting anti-viral ISGs, which restricts multiple viral pathogens with diverse mechanisms. Viperin is upregulated early in some bacterial infections, and using the intracellular bacterial pathogen, *S. flexneri*, we have shown for the first time that viperin inhibits the intracellular bacterial life cycle. *S. flexneri* replication in cultured cells induced a predominantly type I interferon response, with an early increase in viperin expression. Ectopic expression of viperin limited *S. flexneri* cellular numbers by as much as 80% at 5hrs post invasion, with similar results also obtained for the intracellular pathogen, *Listeria monocytogenes*. Analysis of viperins functional domains required for anti-bacterial activity revealed the importance of both viperin’s N-terminal, and its radical SAM enzymatic function. Live imaging of *S. flexneri* revealed impeded entry into viperin expressing cells, which corresponded to a loss of cellular cholesterol. This data further defines viperin’s multi-functional role, to include the ability to limit intracellular bacteria; and highlights the role of ISGs and the type I IFN response in the control of bacterial pathogens.

## Introduction

The role of type II interferon (IFN-γ) in the control of bacterial infection is well established, however the role of both type I and III IFN, and their related interferon stimulated gene products, in a protective host anti-bacterial response remains controversial^1,2^. There are three distinct interferon families, each of which has varying roles in the restriction of viral and bacterial pathogens; Type I IFN which is produced by most cells in the body, type II IFN produced mainly by T cells and natural killer (NK) cells, and lastly type III IFN, which has restricted activity, due to the fact that its receptor is limited mainly to epithelial cells^3,4^. Both type I and III IFN’s have been shown to have a prominent role in the restriction of viral pathogens, inducing the upregulation of hundreds of interferon stimulated genes (ISGs), that create an anti-viral state in the infected cell as well as neighbouring uninfected cells; however, type I IFN (IFNα/β) expression has often been demonstrated to favour bacterial pathogenesis^2^, and very little is known about the role of type III IFN in control of an anti-bacterial response (reviewed in^1^). There has however been a small number of pathogenic bacteria, including *S. pneumoniae*, *E. coli*, *H. pylori* and *S. pyogenes*, for which type I IFN expression has been demonstrated to play a protective role in mouse models of infection^5–8^. Additionally, type I IFNs are also able to restrict the entry of the intracellular bacterial pathogens *Shigella flexneri* and *Salmonella enterica*, but the mechanisms involved remain unknown^9,10^.

Interferon stimulated genes (ISGs) are host effector molecules whose expression is induced via activation of the JAK-STAT pathway following expression of IFN. While the role of many ISGs are uncharacterised, they have been best described in reference to their ability to limit viral infection^11^. It has previously been demonstrated that bacterial pathogens are also able to induce the expression of multiple type I IFN induced genes^12^, and more recent work has shown that a small handful of these ISGs can have direct anti-bacterial effects. The anti-viral IFITM proteins (Interferon induced transmembrane proteins) have been shown to restrict *Mycobacterium tuberculosis* intracellular growth via endosomal maturation pathways, and the interferon inducible GTPases, IRG (immunity related GTPase) and GBP’s (guanylate binding proteins) can specifically target certain intracellular bacteria, destroying their vacuolar compartment required for replication^13,14^. Additionally, the chemokine CXCL10, also an ISG, has recently been demonstrated to induce bacterial killing of *Bacillus anthracis* via membrane disruption, and more recent work involving a cell-based screen of multiple ISGs against *Listeria monocytogenes*, demonstrated that Trim14 was able to restrict intracellular growth of *L. monocytogenes* via a transcriptionally independent manner, however the exact mechanisms are yet to be uncovered^15,16^.

Viperin is one of the broadest acting anti-viral ISGs and has been described to restrict the life cycles of multiple viral families (reviewed in^17,18^), as well as augment both the TLR7/TLR9 and dsDNA response to viral infection^18,19^. Viperin has previously been shown to also be upregulated early during bacterial infection^19–22^, however its role in the restriction of bacterial pathogens remains unknown. Viperin is a 42 kDa, interferon inducible protein that is highly evolutionarily conserved^17,23^, and is induced early in viral infection through both interferon dependent and independent mechanisms^24–29^. It is localised to both the endoplasmic reticulum, as well as the lipid droplet via its N-terminal amphipathic helix^30–32^, and is a member of the radical SAM enzyme family^33^. Viperin is known to directly restrict viral replication of the Flaviviridae family members, HCV, Dengue, Zika, West Nile virus and TBEV^30,34–39^ through interactions with both host and viral proteins, and has also been shown to restrict the egress of HIV, influenza and RSV^40–42^. Viperin’s capacity to inhibit the life cycles of multiple viruses with distinct mechanisms, and its induction upon bacterial infection, poses the question of whether it is able to also restrict the life cycle of intracellular bacteria. Using *S. flexneri* as a model of intracellular bacterial infection, we show that *S. flexneri* is able to induce expression of viperin. Furthermore, loss of viperin was shown to enhance intracellular bacterial levels, and its ectopic expression was demonstrated to restrict bacterial entry *in vitro*, which coincided with reduced cellular cholesterol; demonstrating a further protective role for type I IFN inducible genes in control of intracellular bacteria.

## Results

### *S. flexneri* infection of cells *in vitro* predominantly induces a type I IFN response

**Figure 1:**
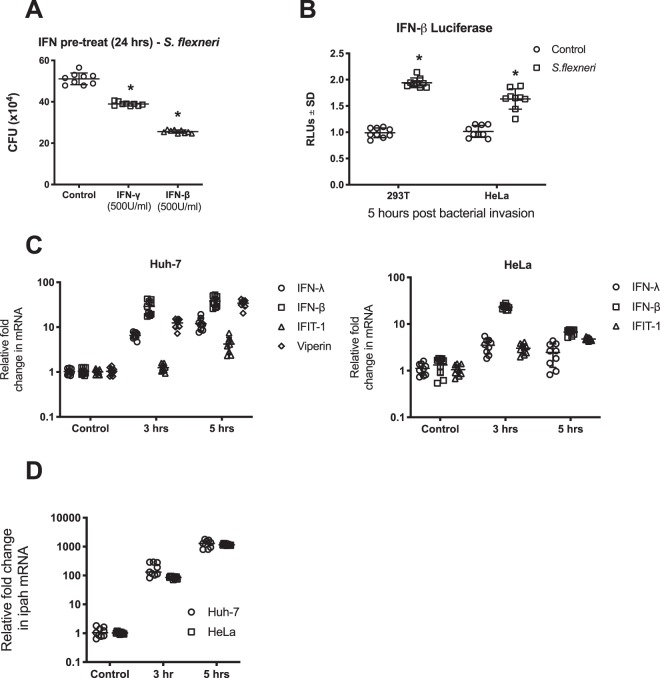
*S. flexneri* induces both a type I and type III IFN response upon invasion of epithelial cells. (**A**) HeLa cells were pre-treated with IFN 24 hours prior to bacterial invasion, and CFU counts performed at 5 hours post infection; p < 0.0001. (**B**) HeLa and 293T cells were transfected with an IFN-β luciferase promoter construct and associated controls 24 hrs prior to *S. flexneri* invasion; cells were harvested for luciferase quantitation 5 hours following invasion. *S. flexneri* invasion of Huh-7 and HeLa cells was performed, and either RNA extracted at both 3 and 5 hours following infection to quantitate (**C**) mRNA from IFN and selected ISGs or (**D**) the constitutively expressed *ipaH* gene from *S. flexneri* via real-time PCR. All graphs represent the mean and standard deviation of three independent experiments performed in triplicate, *p < 0.0001.

Previous reports have demonstrated that both type I and type II interferon can restrict *S. flexneri* growth *in vitro and in vivo*^10,43^, but there is limited work assessing the ability of these intracellular bacteria to induce interferon expression following cellular invasion. To confirm the ability of interferon to limit infection of *S. flexneri* into HeLa cells, we initially pre-treated cells with type I and II interferon for 24 hours prior to invasion assays. As can be seen in Fig. 1A, *S. flexneri* infection was significantly inhibited by up to 50% with IFN-β treatment (Type I IFN), and to a lesser extent with IFN-γ (Type II IFN) pre-treatment (24%).

We next wanted to assess whether *S. flexneri* infection itself was able to induce activation of IRF3, to stimulate the IFN- β promoter *in vitro*. 5 hours post *S. flexneri* invasion of both HeLa and 293 T cell lines, a significant activation of the IFN-β promoter was observed (Fig. 1B); that was supported by increased expression of both interferon- β and -λ at the mRNA level (Fig. 1C). The regulation of IFN and the interferon stimulated genes (ISGs) IFIT1 and viperin, was assessed in the epithelial cell lines, HeLa and Huh-7 cells, with differences observed in gene induction between these two cell lines (Fig. 1C), despite there being no significant difference in the levels of bacteria present in each at both time points (Fig. 1D). Both Huh-7 and HeLa cells predominantly produced type I interferon in response to *S. flexneri* infection, with peak expression of both type I and III IFN seen at 5 hours post invasion in Huh-7 cells. Interestingly this was not mirrored in the HeLa cells, which showed a decrease in type I IFN between 3 and 5 hours post invasion, and an overall lack of type III induction. Huh-7 cells also demonstrated a greater increase in the ISG IFIT1, than HeLa cells, likely due to the enhanced IFN production overall in this cell line. Viperin expression was also assessed in the Huh-7 cells (HeLa cells do not regulate viperin^41^), and was shown to be markedly increased as early as 3 hours post bacterial invasion, with its earlier induction than IFIT1 potentially due to its ability to be directly regulated independently of IFN, via a direct IRF3 mediated mechanism^26^.

### Viperin inhibits both *S. flexneri* and *L. monocytogenes* cellular infection *in vitro*

**Figure 2:**
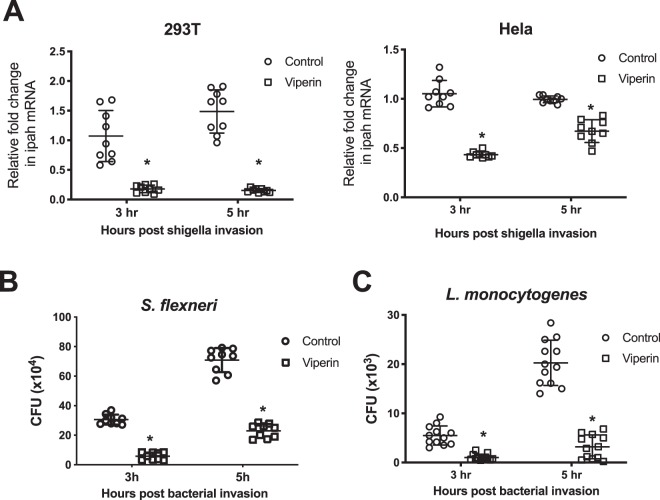
Viperin expression inhibits intracellular bacteria infection of cells *in vitro*. (**A**) Viperin was transiently expressed 24 hours prior to invasion with *S.flexneri* in 293T and HeLa cells, and RNA harvested at the indicated times; graphs represent the mean and standard deviation of three independent experiments performed in triplicate. 293T cells were transiently transfected with viperin 24 hours prior to invasion with either (**B**) *S. flexneri*, or (**C**) *L. monocytogenes*, and CFU counts performed at the indicated times. Graphs represent the mean and standard deviation of three independent experiments performed in triplicate for *S. flexneri* and in quadruplicate for *L. monocytogenes*, *p < 0.0001.

Viperin and other ISGs are known to be upregulated early in response to bacterial infection^22^, and as previously discussed we have shown that Huh-7 cells upregulate viperin as early as 3 hours post *S. flexneri* invasion (Fig. 1C). In order to assess the ability of viperin to limit bacterial infection we first expressed viperin transiently in both 293T and HeLa cells, 24 hours prior to invasion with *S. flexneri*. Viperin expression significantly decreased the levels of intracellular bacteria at both 3 hours and 5 hours post invasion by 82% and 87% respectively in 293T cells, and 57% and 33% respectively in HeLa cells (Fig. 2A); this was accompanied by a significant drop in intracellular colony forming units at both time points in 293T cells (80% and 67% respectively)(Fig. 2B). The differences in viperin’s ability to limit *S. flexneri* infection in the 2 different cell types is most likely due to the transfection efficiency and corresponding viperin expression, and as such 293T cells were used for further experimental analysis. Interestingly, the ability of viperin to inhibit bacterial infection was not limited to only *S. flexneri*, with inhibition rates of 84% and 85% also observed at 3 and 5 hours respectively post invasion of 293T cells with *L. monocytogenes* (Fig. 2C); indicating that viperin’s ability to limit the intracellular bacterial levels of *S. flexneri* may be a more general inhibition of intracellular bacteria, rather than a specific inhibition.

**Figure 3:**
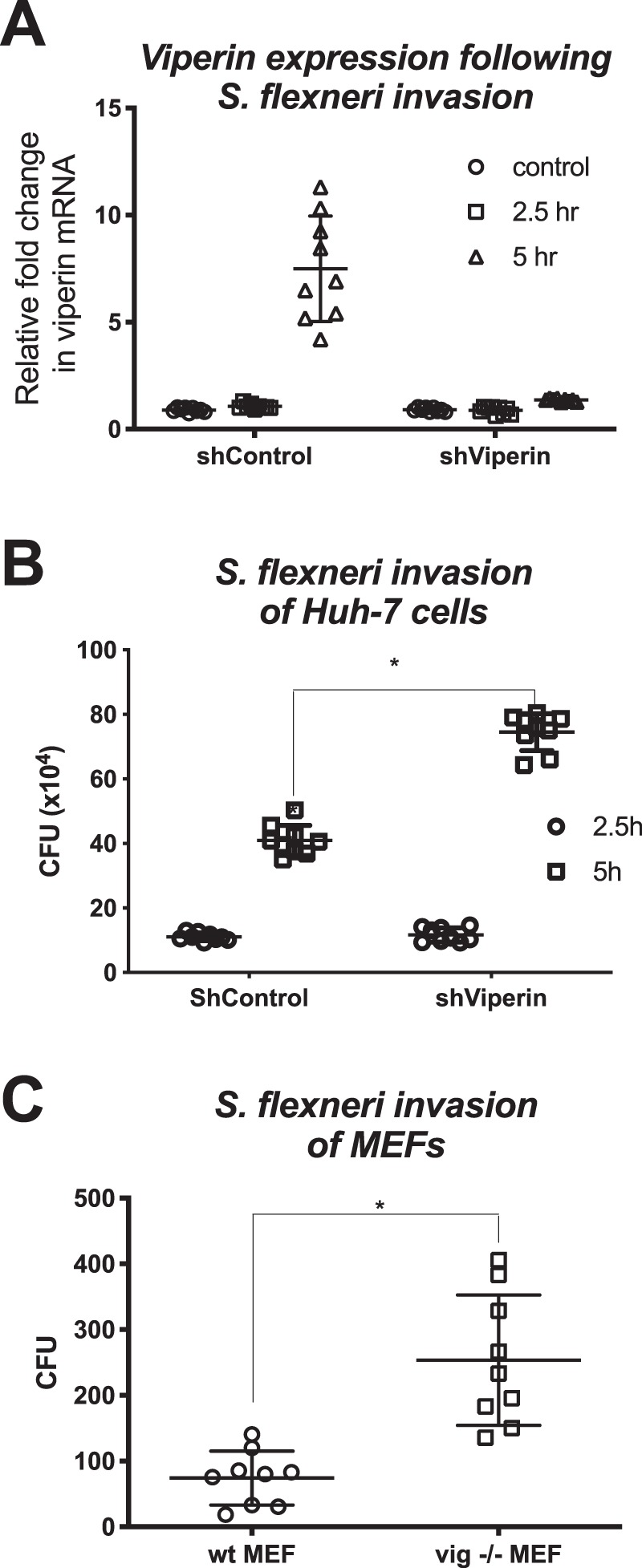
Loss of viperin enhances *S. flexneri in vitro*. (**A**) Viperin shRNA or control shRNA expressing Huh7 cells were invaded with *S. flexneri* and either RNA collected for real-time analysis or (**B**) cell lysate collected for CFU counts, at the indicated time points. (**C**) Primary MEFs harvested from either wild-type or viperin KO mice were invaded with *S. flexneri* and cell lysates collected for CFU counts at 5 hours post invasion. Graphs represent the mean and standard deviation of three independent experiments performed in triplicate, *p < 0.0001.

To assess the impact of loss of viperin on *S. flexneri* infection *in vitro*, we utilised Huh-7 cells with impaired viperin expression as we have discussed previously^30^. Initial infection of the Huh-7 cells demonstrated that the control cells (shControl) were able to induce viperin mRNA expression at 5 hours post *S. flexneri* invasion, while no viperin expression was detected at any time point in the Huh-7 shViperin cell line (Fig. 3A). This induction of viperin coincided with a decreased ability of *S. flexneri* to infect shControl Huh-7 cells at 5 hours post invasion by approximately 45% (Fig. 3B), demonstrating that viperin expression impacts *S. flexneri* infection *in vitro*. To further confirm the role of viperin in the control of *S. flexneri*, we next assessed the ability of the bacterium to infect viperin knock-out murine embryonic fibroblasts (MEF’s) sourced from our previously described CRISPR/cas derived Vip -/- del32 strain in comparison to wild-type fibroblasts^38^. As can be seen in Fig. 3C, MEFs absent in viperin expression had a 3.4 fold higher rate of *S. flexneri* infection than wild-type MEFs, confirming the impact that viperin has on the outcome of successful *S. flexneri* infection of cells *in vitro*.

### Viperin’s enzymatic activity impacts its ability to limit *S. flexneri* infection *in vitro*

**Figure 4:**
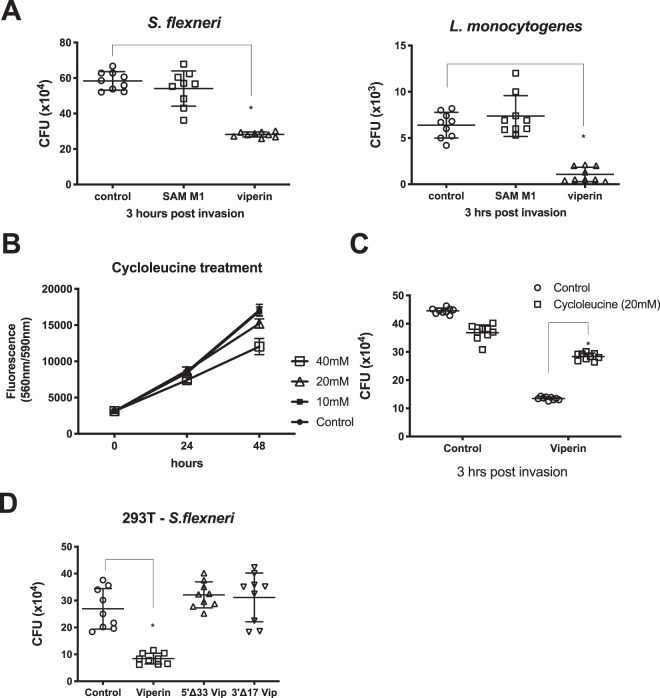
Viperin requires its radical SAM domain to limit intracellular bacterial numbers. (**A**) 293T cells were transiently transfected with either a viperin wt expressing plasmid, or a SAM domain viperin mutant 24 hours prior to invasion with either *S. flexneri* or *L. monocytogenes*; cell lysates were harvested for CFU counts at 3 hours post invasion. (**B**) 293T cells were treated with the indicated concentrations of cycloleucine, and a cell viability assay performed at the indicated time points. (**C**) Transfection of 293T cells with either a control or a viperin expressing plasmid was performed 24 hours prior to cycloleucine or control treatment for 24 hours. Cells were then invaded with *S. flexneri* for 3 hours prior to cell lysate harvesting for CFU counts. (**D**) 293T cells were transfected with either a control, viperin wt, or viperin truncation mutants 24 hours prior to invasion with *S. flexneri* for 3 hours. Graphs represent the mean and standard deviation of three independent experiments performed in triplicate, *p < 0.0001.

Viperin is a radical SAM enzyme, that has recently been demonstrated to catalyse the conversion of CTP to the novel molecule, ddhCTP (3′-deoxy-3′,4′-didehydro-CTP), which acts a chain terminator of RNA dependent RNA polymerases of selected members of the Flaviviridae viral family^39^. However, viperin has antiviral activity beyond its enzymatic activity^17^, and this prompted us to determine whether viperins’ enzymatic activity was required for its ability to limit intracellular bacteria. Viperin mutants harbouring point mutations of conserved cysteine residues in the S1 domain (SAM M1^30^) were expressed transiently in 293T cells in parallel with wild-type viperin, and *S. flexneri* and *L. monocytogenes* invasion assessed. The ability of SAM M1 viperin to limit both *S. flexneri* and *L. monocytogenes* was completely abrogated in cells expressing the SAM M1 mutant (Fig. 4A). To further analyse the ability of the radical SAM enzymatic domain of viperin to play a role in limiting intracellular bacterial infection, we performed invasion assays in the presence of cycloleucine, a known inhibitor of cellular SAM synthesis, and an inhibitor in general of radical SAM enzymatic activity^44^. Cycloleucine did not impact cell viability at concentrations at or lower than 20 mM when added to the culture media of 293T cells (Fig. 4B), neither did it have a significant impact on *S. flexneri* infection of 293T cells 3 hours post invasion (Fig. 4C). However, 293T cells pre-treated with cycloleucine prior to *S. flexneri* invasion, showed a significant difference in intracellular bacterial numbers 3 hours post invasion, with viperin expression decreasing bacterial counts by approximately 70%, but only 13% in the presence of cycloleucine; reinforcing the fact that viperin’s radical SAM enzymatic activity is important for its ability to limit *S. flexneri* infection of cells *in vitro*.

A number of domains/regions of importance have been classified within viperin, including its N-terminal amphipathic helix, which we and others have previously shown to localise viperin to lipid droplets and the endoplasmic reticulum^30,32^, as well as its C-terminal region which has been shown to be vital in its ability to restrict some viral infections (reviewed in^17^). To assess the importance of these two domains, truncated viperin mutants were expressed transiently prior to invasion of 293T cells with *S. flexneri*. Both the amphipathic helix and the C-terminal region of the viperin protein were found to be vital in the ability of viperin to limit *S. flexneri* bacterial counts at 3 hours post bacterial invasion, with the truncated viperin mutants showing no significant difference in bacterial counts from the controls (Fig. 4D).

### Viperin limits initial entry of *S. flexneri in vitro*

**Figure 5:**
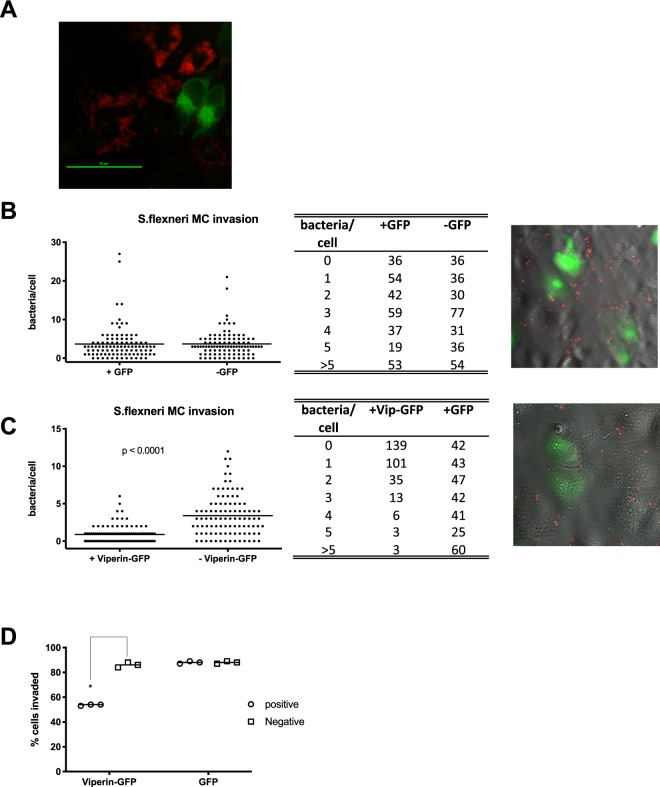
Viperin expression inhibits the entry of *S. flexneri in vitro*. (**A**) Huh-7 cells transiently expressing viperin-GFP where invaded with an *S. flexneri* strain expressing mCherry and imaged 5 hours post invasion. (**B**,**C**) Huh-7 cells transiently expressing either viperin-GFP or GFP alone, where invaded with an *S. flexneri* strain expressing mCherry for 15 minutes prior to fixation and imaging on a Nikon TiE inverted microscope; Whole fields were counted at random and assessed for the number of intracellular *S. flexneri* per cell (n = 300 cells per group, counted over 3 biological replicates), as well as (**D**) categorised as either *S. flexneri* infected or not, to assess percentage of cells invaded by the bacteria. *p < 0.0001.

In order to gain insight into the life cycle stage at which viperin inhibits *S. flexneri*, we used microscopy to gain a broader view of the potential interactions between viperin and bacterially infected and uninfected cells *in vitro*. Experiments investigating infection of an mCherry expressing *S. flexneri* (MLRM107,^45^) into viperin pre-transfected Huh-7 cells, displayed an almost complete exclusion of bacteria 3 hours post invasion, in many cells expressing viperin (Fig. 5A). In order to quantitate exclusion at an earlier time point, Huh-7 cells were transfected with either a viperin-GFP expression plasmid, or GFP only, 24 hours prior to invasion of the cells with mCherry labelled *S. flexneri*. Cells were fixed 15 minutes post infection, and then visualised using microscopy. Whole fields were imaged, and cells counted at random until a minimum of 300 viperin-GFP or GFP positive and negative cells were assessed for the number of intracellular *S. flexneri* per cell. In cells expressing GFP only, we were able to visualise on average, 3.66 bacteria per Huh-7 cell, in comparison to 3.68 bacteria per cell in GFP negative cells (Fig. 5B), demonstrating that there was no significant difference between the ability of *S. flexneri* to invade Huh-7 cells whether or not they were expressing the GFP protein. Interestingly, in the presence of viperin-GFP expression, the average number of *S. flexneri* present per cell was significantly reduced to 0.89 bacteria, in comparison to 3.41 bacteria per cell in the viperin-GFP negative cells (Fig. 5C), equating to an approximate 3.8 fold reduction in bacterial invasion per cell. When taking into account the actual number of cells that contained at least one bacterium, irrespective of the total amount of bacteria per cell, we can see that in cells expressing viperin, approximately 54% of cells were positive for early bacterial invasion, in comparison to approximately 86–88% of cells in the presence or absence of GFP (Fig. 5D), indicating that the Huh-7 cells expressing viperin where overall less permissive for *S. flexneri* invasion at early time points following infection.

### Viperin expression impacts cholesterol levels *in vitro*

**Figure 6:**
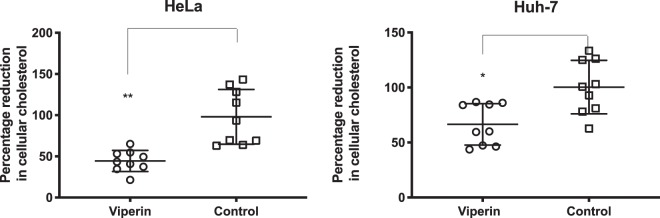
Viperin expression lowers cellular cholesterol levels *in vitro*. Huh-7 and HeLa cells transiently expressing either viperin or transfected with a control plasmid were assessed 24 hours post transfection for their cellular levels of cholesterol. Graphs represent the mean and standard deviation of three independent experiments performed in triplicate, *p < 0.05, **p < 0.001.

*S. flexneri* intracellular invasion is known to be supported by an interaction between host cell CD44 and the bacterial invasin IpaB, that occurs on lipid rafts enriched in cholesterol and sphingolipids^46^. Viperin expression has previously been identified to alter the fluidity of lipid rafts *in vitro*, and to reduce cholesterol levels in BHK cell lines^41,47^. We next assessed the ability of transient viperin expression to lower the levels of cholesterol in both HeLa and Huh-7 cells. As can be seen in Fig. 6, the expression of viperin was able to significantly lower the levels of cholesterol in both HeLa and Huh-7 cells by approximately 55% and 33% respectively, with the difference likely reflecting the transfection efficiency of these two cell types.

## Discussion

The role of the classical anti-viral cytokines, type I (IFN-α,β) and type III IFN (IFN-λ), and the interferon stimulated genes that they upregulate, in the control of bacterial infection remains controversial^1,2^. Multiple extracellular and intracellular bacterial species are known to upregulate these interferons following infection of host cells, with the activation of type I IFN being mainly via STING dependent mechanisms, and through bacterial LPS activation of TLR4, with a small number of bacterial species being shown to also induce type I IFN via engagement of NOD1/NOD2^48^. The induction of type III IFN by bacteria is less well studied, but is also induced by a range of both intracellular and extracellular pathogens. Both TLR7 and MAVS activation have been proven to be crucial for type III IFN induction by *Borrelia burgdorfei* and *Listeria monocytogenes* infection respectively, with the mechanisms of induction by other bacterial species not being well characterised to date^1^. Despite the knowledge that bacterial infection of a host induces these cytokines, their respective ability to restrict bacterial pathogenesis and directly inhibit the bacterial life-cycle remains contentious.

Hundreds of interferon stimulated genes are induced following activation of the JAK-STAT pathway by type I and III IFNs, with only a small handful of these to date being demonstrated to directly inhibit the life-cycle of some bacterial species. Viperin is an interferon stimulated gene that is upregulated via multiple pathways, including via IFN-independent means^17^. It belongs to the radical SAM family, which are enzymes that bind iron-sulphur clusters to mediate their reactions; and interestingly is one of a handful of mammalian radical SAM enzymes, with the majority of known radical SAM members belonging to bacterial family members^49^. Viperin is known to be induced following cellular invasion of the intracellular bacterial pathogens, *Mycobacterium tuberculosis*, *Listeria monocytogenes* and *Salmonella typhimurium*^19,21^. Additionally, in a fish muscle model of bacterial infection, *Vibrio vulnificus* colony forming units were significantly reduced when a plasmid expressing viperin was electroporated into fish muscle prior to bacterial infection, although the exact mechanisms behind viperin’s potential anti-bacterial activity were not elucidated^50^. Here we show that *S. flexneri* infection of cultured cells *in vitro* is significantly hampered by the presence of IFN, and that cellular infection by the bacteria is able to upregulate viperin expression in epithelial cell lines *in vitro* (Fig. 1C). Interestingly, *S. flexneri* appeared to illicit a more dominant type I IFN response in the two epithelial cell lines tested, which coincides with previous work demonstrating that *S. flexneri* is a weak inducer of Type III IFN species in epithelial cells^51^.

Both ectopic viperin expression and loss of viperin expression significantly impacted the growth of the gram-negative pathogen, *S. flexneri* in multiple cell lines *in vitro*, and this observation was also extended to the gram-positive intracellular pathogen, *L. monocytogenes*. The ability of viperin to impact the growth of these intracellular bacteria relied on its radical SAM enzymatic activity, as well as the presence of its N-terminal amphipathic helical domain, and its extreme C-terminal domain (Fig. 4). Viperin’s N-terminus contains both an amphipathic helix which is responsible for both its localisation to the lipid droplet, and the endoplasmic reticulum^30^, as well as a binding region for one of its iron sulphur targeting factors, CIA2A, with the others (CIA1/2B) binding to its C-terminal region^52^. Viperin’s enzymatic activity has recently been elucidated to involve the formation of the novel ribonucleotide, 3’-deoxy-3’, 4’-didehydro-CTP (ddhCTP) via catalysing the conversion of cytidine triphosphate (CTP)^39^. This novel compound was shown to act in an anti-viral manner by restricting polymerase activity of a small handful of members from the flavivirus genus, however its enzymatic activity has been shown to be redundant for its ability to limit other viral family members^30,39^. It is clear that viperin is a highly multifunctional protein, and recent work has demonstrated that viperin can also augment the STING dependent dsDNA signalling pathway, with this function being dependent on its co-factor binding sites, and the presence of its radical SAM enzymatic domains, perhaps indicating that viperin’s radical SAM enzymatic activity has multiple host cell purposes^53^.

Prior ectopic expression of viperin in epithelial cells *in vitro* appeared to exclude bacterial replication in these cells (Fig. 5A), and subsequent infection assays revealed that the presence of intracellular levels of viperin reduced the numbers of *S. flexneri* in cells by almost 4 fold (Fig. 5C). Viperin has been shown previously to inhibit the egress of influenza, HIV and respiratory syncytial virus virions, with the common mechanism being changes in cholesterol levels, or lipid raft membrane fluidity^40–42^, and here we were able to demonstrate that viperin expression significantly reduces the levels of cholesterol in both Huh-7 and HeLa epithelial cell lines (Fig. 6). Lipid raft cholesterol is essential for entry of *Shigella spp*. into epithelial cells, where its invasin, IpaB, is known to interact with hyaluronan receptor, CD44, and ultimately cause redistribution of plasma membrane cholesterol to the sites of bacterial invasion^46,54^. Given’s viperin’s previously known ability to limit viral egress via interruption of cholesterol in membranes, in particularly altering plasma membrane fluidity^41^, it is likely that viperin expression might also be disrupting *S. flexneri* entry through the diminished presence of plasma membrane cholesterol, however further studies need to be performed to elucidate the exact mechanism that viperin utilises to impede entry of *S. flexneri*, and whether it alters the ability of *S. flexneri* to utilise cellular entry receptors. Another limitation of this study, is the inability to compare bacterial growth and spread kinetics between a viperin competent and a deficient model system, due to the very low baseline of the protein, and the time it takes to be induced following bacterial infection (Fig. 3A). However, we would hypothesise that in a spreading infection a bystander effect would likely occur, where interferons produced by an *S. flexneri* infected cell, would upregulate viperin in surrounding as yet uninfected cells, and potentially impact bacterial entry.

Although the potential mechanisms at play in the control of bacterial pathogenesis by type I and III IFN remain in their infancy, recently it has been shown that type III IFNs can reinforce epithelial barriers during bacterial infection, and prevent invasion from intracellular bacteria such as *Shigella spp*.^55^, although the ISGs responsible for this effect remain unknown. Despite similar signalling pathways, type I and III IFNs are known to selectively induce ISGs, with differential kinetics of expression, as well as an altered ISG expression profile depending on cell type^56^. Viperin is known to be expressed in intestinal epithelial cells, and is a dominantly expressed protein in multiple cell types, regardless of its induction by type I or III IFN^17,57,58^. Here we present data for the first time that the interferon stimulated gene, viperin, not only exhibits broadly acting anti-viral capabilities, but is also able to restrict the infection of the intracellular pathogen, *S. flexneri*. This work paves the ways for future studies investigating the role of other multifunctional ISGs and their ability to restrict bacterial pathogens, and possibly help unravel the role of both type I and III IFNs in direct control of bacterial infection.

## Methods

### Cells and bacterial stocks

Huh-7 human hepatoma cells, which are a well differentiated hepatocyte-derived carcinoma cell line (a kind gift from Stanley Lemon, The University of Texas Medical Branch); HeLa human epithelial cells which a cervical carcinoma cell line (laboratory stock gifted from the Institute of Medical and Veterinary Science, Adelaide), and 293T cells, which are a derivative of the 293 human embryonic kidney cell line (a kind gift from Murray Whitelaw, University of Adelaide), were maintained as previously described^59^, and at 37 °C with 5% CO_2_ in DMEM containing 10% FCS, penicillin and streptomycin. Huh-7 shControl cells, and the shViperin cells were maintained as above, and are described in^30^. Murine embryonic fibroblasts (MEF’s) were isolated from both wild-type and viperin knock-out mice and maintained as previously^38^. The isolation of all MEFs was carried out in accordance with the relevant guidelines and regulations of the National Health and Medical Research Council Australian Code of Practice for the Care and Use of Animals for Scientific Purposes, and approved by the University of Adelaide Animal Ethics Committee. The *S. flexneri* strains 2457 T and MLRM107^45^, were grown from a Congo Red positive colony as described previously^60^, and cultured in Luria Bertani (LB) broth and on LB agar. *L. monocytogenes* was cultured in Luria Bertani (LB) broth and on LB agar. Bacteria were grown in LB media for 16 hours, prior to being subcultured 1:20, and grown to mid-exponential growth phase for 2 hours at 37 °C with aeration. MLRM107 cultures were supplemented with tetracycline (10 μg/l). Cell viability assays were performed using a Cell Titre Blue assay kit (Promega) as previously^61^.

### *S. flexneri* invasion and detection

Cell lines were seeded at 8 × 10^4^ in 12 well plates, 24 hours prior to invasion with *S. flexneri* 2457 T. Cells were washed twice in PBS, followed by one wash in complete DMEM lacking antibiotics. Mid-exponential phase *S. flexneri* (4.8 × 10^7^ bacteria per well) were added to the cells in a 200 μl volume of complete DMEM without antibiotics, and the plates spun at 2,000 g for 7 minutes. After a one hour incubation at 37 °C with 5% CO_2_, the cells were washed 3 times with PBS, and the media replaced with DMEM containing 40 μg/ml gentamicin. At the indicated time points cells were washed three times with PBS and were either harvested for RNA extraction, or lysed with 0.1% (v/v) Triton X-100 in PBS for 5 minutes and the bacteria enumerated on LB plates. All experiments were repeated in at least triplicate.

### RNA extraction and RT-PCR

Total cellular RNA was isolated from cells using Trizol (Invitrogen, Calsbad, USA), DNase treated and quantitated by spectrophotometry. cDNA synthesis and subsequent real-time PCR was performed on an ABI 7000 as previously described for the detection of IFN’s, ISGs and the house keeping gene, RPLPO mRNA^62^. Primer sequences used to detect *S. flexneri* iPAH mRNA were 5′-TTGGTCGCCCTACCTTTTCA and 5′-CTCGATAAGCGCAGAGAAATGA.

### Plasmids, transfections and cell stimulants

Viperin wt and mutant plasmids were as described previously^30^. Cell lines were seeded at 7 × 10^4^ into 12 well plates, 24 hours prior to transfection with DNA vectors using Fugene6 (Roche, Penzburg, DEU) as per manufacturer’s instructions, or stimulation with IFN-γ, β and IFN-α (Peprotech, Israel) for 24 hours. All experiments were repeated in at least triplicate.

### Luciferase experiments

Cells were seeded at 7 × 10^4^ in 12 well plates 24 hours prior to transfection with an IFN-β promoter driven luciferase plasmid (pIF(−125/ + 75)lucter (a kind gift from Dr Liu, Royal Children’s Hospital, QLD) and the pRL-TK constitutively expressing renilla luciferase plasmid to control for transfection efficiency. Twenty four hours following transfection, cells were invaded with 4.8 × 10^4^ bacteria per well of *S. flexneri* 2457 T as above, for 5 hours prior to luciferase detection using the Dual-Luciferase Reporter Assay System (Promega, Fitchberg, USA) as per manufacturer’s instructions. Luciferase measurements were taken on a Promega GloMax 96 luminometre. Experiments were performed in triplicate.

### Inhibition of radical SAM enzyme function

To inhibit viperin’s radical SAM enzyme function, cells were treated with cycloleucine (Sigma, St. Louis USA) for 24 hours as previously described^37^, 24 hours post DNA plasmid transfection. Cells were washed twice in PBS, prior to invasion with *S. flexneri* 2457 T for 3 hours, as described above. Cell viability assays were performed using a Cell Titre Blue Assay kit (Promega, Fitchberg, USA) as previously described^61^.

### *S. flexneri* early entry assay

Huh-7 cells were seeded at 2 × 10^4^ cells per well of a 24 well plate, containing a sterile gelatin coated glass coverslip in the bottom. Cells were transfected with either viperin-GFP or GFP alone as described previously^30^, and 24 hours later invaded with 2.4 × 10^7^ bacteria per well of the mCherry fluorescing *S. flexneri* MLRM107 strain. Cells were spun at 2,000 g for 7 minutes and incubated for a further 15 minutes at 37 °C with 5% CO_2_. Cells were then washed vigorously 3 times with PBS, prior to fixation with 4% paraformaldehyde for 20 minutes. Coverslips were mounted, and florescence was visualised using a Nikon TiE inverted microscope (Nikon, Japan). Cells were visualised using a 40x objective, and whole fields counted at random until a minimum of 300 GFP positive and negative cells were assessed for the number of intracellular *S. flexneri* per cell. Experiments were performed in triplicate.

### Assessment of cellular cholesterol levels

Cells were seeded at 6 × 10^4^ cells per well of a 12 well plate 24 hours prior to transient transfection using Lipofectamine 3000, with either a viperin expression plasmid or an empty vector. 24 hours following transfection, cells were harvested and lysed with 0.1% triton X-100. The cholesterol content of the cell lysates was quantified with the cholesterol quantification kit (40006; AAT Bioquest, USA) according to the manufacturer’s specifications. Each condition was performed at least 8 times and on four independent occasions. Fluorescent signal was monitored at Ex/Em = 540/590 nm (cholesterol) using a microplate reader (CLARIOstar® BMG LABTECH).

### Immunostaining and co-localisation studies

Cells were cultured on gelatin coated glass coverslips, fixed in acetone:methanol (1:1) and stored at −20 °C. Slides were washed in PBS, before blocking in 5% (w/v) bovine serum albumin (BSA) in Hanks buffered salts solution (Gibco BRL, NY). Cells were stained using a rabbit anti-mCherry antibody (BioVision, CA) together with a goat anti-rabbit IgG-Alexa 555, secondary antibody (Molecular probes, CA). Bodipy 493/503 (Invitrogen) was prepared as a stock solution of 1 mg/ml in ethanol and used at a 1:1000 concentration PBS. Florescence was visualised using a Nikon TiE inverted microscope (Nikon, Tokyo, Japan).

### Statistical analysis

Mann-Whittney U tests (2 tailed) were utilised to analyse the distributions of two data sets and all experiments were performed a minimum of three times and in at least triplicate. Statistical analysis was performed using PRISM8 (GraphPad software). The data points for all figures can be found in Supplementary Table 1.

## Supplementary information

Supplementary File 1
